# Characteristics of mucin hypersecretion in different inflammatory patterns based on endotypes of chronic rhinosinusitis

**DOI:** 10.1002/clt2.12334

**Published:** 2024-01-22

**Authors:** Zhaoxue Zhai, Liting Shao, Zhaoyang Lu, Yujuan Yang, Jianwei Wang, Zhen Liu, Huikang Wang, Yang Zheng, Haoran Lu, Xicheng Song, Yu Zhang

**Affiliations:** ^1^ Second Clinical Medicine College Binzhou Medical University Yantai China; ^2^ Department of Otolaryngology Head and Neck Surgery, Yantai Yuhuangding Hospital Qingdao University Yantai China; ^3^ Shandong Provincial Clinical Research Center for Otorhinolaryngologic Diseases Yantai China; ^4^ Yantai Key Laboratory of Otorhinolaryngologic Diseases Yantai China

**Keywords:** chronic rhinosinusitis, cytokines, endotypes, immune response, mucin hypersecretion

## Abstract

**Background:**

Chronic rhinosinusitis (CRS) is usually accompanied by mucin hypersecretion that can lead to mucus accumulation and impair nasal mucociliary clearance, thus exacerbating airway inflammation. Abnormal mucin hypersecretion is regulated by different T helper (Th) cytokines, which are associated with different endotype‐driven inflammatory responses. Therefore, it is of great significance to understand how these factors regulate mucin hypersecretion to provide precise treatment strategies for different endotypes of CRS.

**Body:**

Thus far, the most common endotypes of CRS are classified as type 1, type 2, or type 3 immune responses based on innate and adaptive cell‐mediated effector immunity, and the representative Th cytokines in these immune responses, such as IFN‐γ, TNF‐α, IL‐4, IL‐5, IL‐13, IL‐10, IL‐17, and IL‐22, play an important regulatory role in mucin secretion. We reviewed all the related literature in the PubMed database to determine the expression of these Th cytokines in CRS and the role they play in the regulation of mucin secretion.

**Conclusion:**

We believe that the main Th cytokines involved in specific endotypes of CRS play a key role in regulating abnormal mucin secretion, which contributes to better understanding of the pathogenesis of CRS and provides therapeutic targets for airway inflammatory diseases associated with mucin hypersecretion.

## 1 INTRODUCTION

As the main solid component of the airway mucus layer, mucins cooperate with water, ions, and proteins on the airway surface to form the mucus‐ciliary clearance system under physiological conditions. This clearance system serves to resist the pathogen invasion and maintain the integrity and the normal function of nasal mucosal epithelium, which is an important component of the innate immune system.^1^, ^2^ Mucins are divided into two families: secreted and cell‐tethered mucins, respectively. A total of 21 mucin genes have now been identified in humans, and a total of 8 mucins have been detected in chronic sinusitis (CRS) tissues, including 6 secreted mucins (MUC2, MUC5AC, MUC5B, MUC6, MUC7, and MUC8) and two membrane‐bound mucins (MUC1 and MUC4).^3^ However, in chronic nasal diseases such as CRS, excessive mucin secretion can lead to excessive production of mucus and reduced function of the mucocilia clearing system. This can result in a large amount of mucus blocking the airway, damage to the tight connection and integrity of nasal mucosal epithelial cells, and aggravation of a pre‐existing airway inflammation.^4^, ^5^ Therefore, a mucin secretion imbalance plays an important role in the occurrence and development of CRS. CRS not only significantly affects a patient's quality of life, but also aggravates the mental and psychological burden of a patient, leading to anxiety and depression, and causing a huge economic burden to patients and society.^6^, ^7^ Research on the mechanism of mucin secretion imbalance will be of great significance in further revealing the pathogenesis and optimal clinical treatment of CRS.

The phenotyping of CRS based on the presence or absence of nasal polyps has been widely used to study the clinical manifestations and prognosis of CRS.^8^, ^9^, ^10^ As our understanding of the pathomechanism of CRS has progressed, we increasingly believe that CRS is not a dichotomous or one‐dimensional linear disease spectrum, but rather a multidimensional and heterogeneous disease. This has led to the concept of CRS endotyping, by which CRS is classified by studying the roles of specific molecules or cells in the occurrence and development of CRS based on its physiological pathogenesis. Thus far, the most common endotypes of CRS are classified as type 1, type 2, or type 3 immune responses, as based on innate and adaptive cell‐mediated effector immunity.^11^, ^12^ Type 1 immune responses are Th1 cell‐ and monocyte‐macrophage centered responses. It has been suggested that CRS without nasal polyps (CRSsNP) displays a Th1 inflammatory pattern,^13^ which is mainly characterized by the release of cytokines from dendritic cells and macrophages after exposure to antigens. Cytokine release promotes the differentiation of naive T cells into Th1 cells that produce Interferon gamma (IFN‐γ) and tumor necrosis factor‐α (TNF‐α).^14^ In CRS, type 2 immune responses are typically characterized by the production of cytokines such as interlukin (IL)‐ 4 (IL‐4), IL‐5, IL‐13, and IL‐10 by group 2 innate lymphoid cells (ILC2) and Th2 cells. These cytokines subsequently induce the development of immune responses, which are usually accompanied by an increased number of eosinophils and total IgE levels.^11^, ^15^ CRS can be divided into categories of moderate and severe type 2 inflammation based on the levels of Th2‐related cytokines.^16^ Type 3 immune responses in CRS are mainly characterized by IL‐17A and IL‐17F produced from Th17 cells and ILC3.^17^ There were more Th17 cells in CRS with nasal polyps (CRSwNP) patients than in CRSsNP patients, and that Th17 cells could induce tissue damage by the production of IL‐17 and IL‐22, thus promoting the occurrence and development of CRSwNP.^18^, ^19^

Abnormal mucin secretion in CRS is closely related to an imbalance of immune responses mediated by the typical Th cytokines mentioned above, such as IFN‐γ, TNF‐α, IL‐4, IL‐5, IL‐13, IL‐17, and IL‐22. Therefore, the mechanisms that trigger abnormal mucin secretion are different in different endotypes of CRS and can be determined by inflammatory signatures. Mucins have shifted from being regarded as biomarkers to serving as mechanistic components in CRS. Thus, investigating the roles that inflammatory cytokines play in abnormal mucin secretion in different endotypes of CRS is helpful for gaining a better understanding of the mechanism that regulates mucin hypersecretion. Here, we summarize the mechanisms involved in the regulation of mucin secretion by Th cytokines associated with different immune responses in CRS.

## 2 CHARACTERISTICS OF MUCIN HYPERSECRETION IN DIFFERENT ENDOTYPES OF CRS

### 2.1 Regulation of mucin by Th1 cytokines associated with a type 1 immune response

#### 2.1.1 IFN‐γ and mucin

**FIGURE 1:**
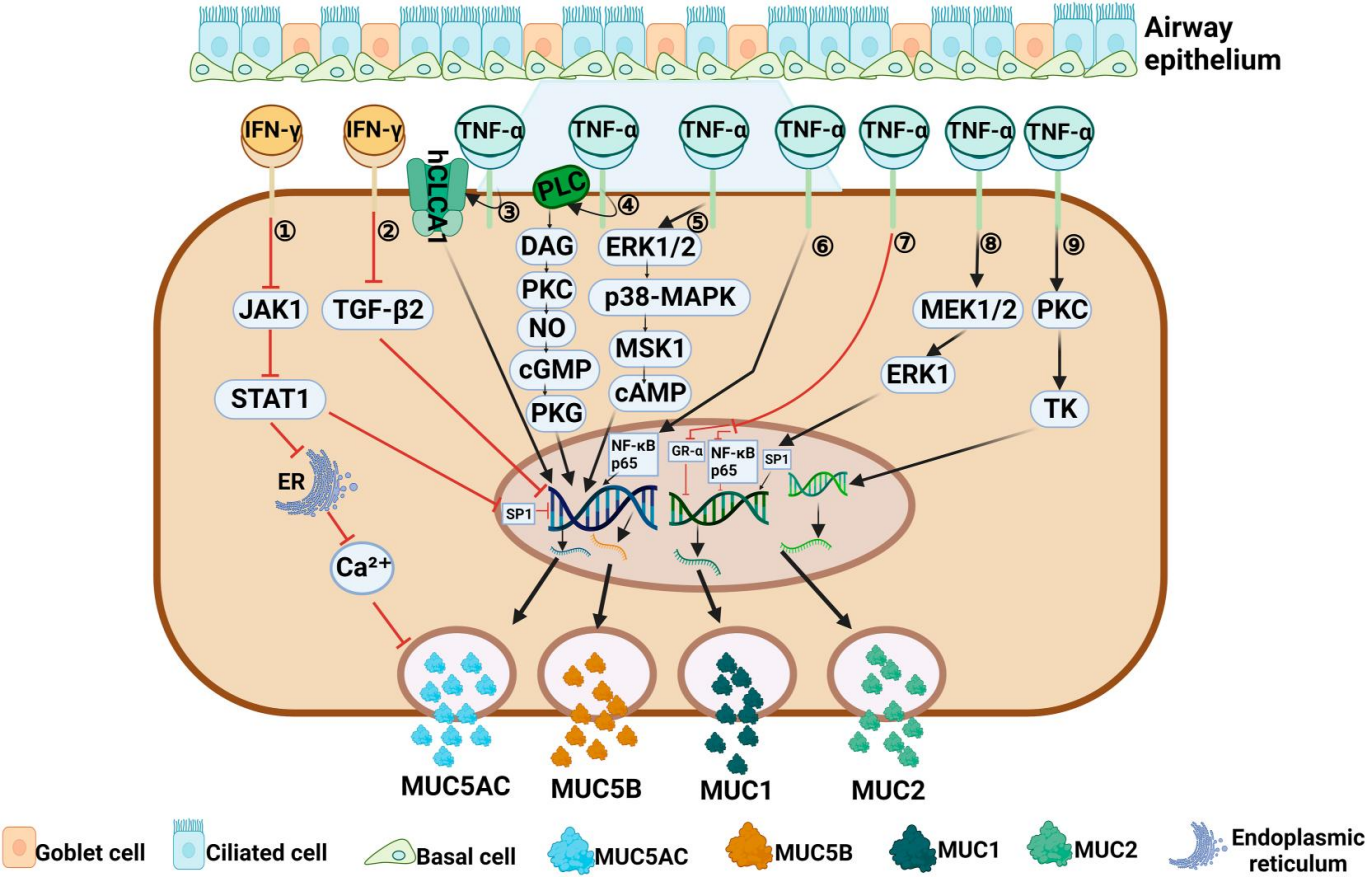
Regulation of mucin by Th1 cytokines associated with a type 1 immune response. (1) IFN‐γ can inhibit the release of Ca^2+^ from the endoplasmic reticulum through the JAK1/STAT1 signaling pathway,^29^ and can also block the binding of Sp1 to MUC5AC promoter region through the JAK1/STAT1 pathway,^27^ thus inhibiting the expression of *MUC5AC* gene. (2) IFN‐γ can directly inhibit MUC5AC expression through TGF‐β2 signaling pathway.^24^ (3) TNF‐α can significantly up‐regulate the expression of MUC5AC by hCLCA1.^46^, ^47^, ^48^ (4, 5) TNF‐α induces the secretion of MUC5AC and MUC5B by activating two signaling pathways: PLC→DAG→PKC→NO→cGMP→PKG,^54^ ERK1/2→p38MAP→MSK1→cAMP.^39^ (6) TNF‐α can promote the expression of *MUC5AC* gene by inducing nuclear translocation of NF‐κB p65 subunit.^37^ (7) TNF‐α can impaired GR‐α nuclear translocation and inhibit the expression of NF‐κB p65, thus inhibiting the expression of *MUC1* gene.^51^ (8) TNF‐α can induce *MUC1* gene transcription by activating the MEK1/2/ERK1/Sp1 pathway.^52^, ^53^ (9) TNF‐α can induce *MUC2* gene transcription by activating the PKC/TK pathway.^40^, ^41^

IFN‐γ is secreted by cytotoxic T cells, Th1 cells, and natural killer cells, and is a typical Th1 cytokine. IFN‐γ levels are higher in certain types of CRS, such as non‐eosinophilic CRS (non‐ECRS), CRS with aspirin intolerance, and CRS with viral infection.^20^, ^21^, ^22^ IFN‐γ acts by binding to its receptor, which is widely expressed on the surface of cells other than red blood cells. IFN‐γ promotes epithelial‐mesenchymal transition (EMT) in human nasal mucosal epithelial cells via the JAK1/2‐STAT1‐ICSBP‐p38 and ERK1/2 signaling pathways.^23^ At present, most studies have suggested that IFN‐γ might negatively regulate mucin secretion (Figure 1). In mouse primary tracheal epithelial cells, IFN‐γ reduces mucin production in two ways. First, IFN‐γ can directly inhibit MUC5AC expression via the TLR2‐IFN‐γ‐TGF‐β2 signaling pathway^24^; second, IFN‐γ can indirectly reduce MUC5AC production by inhibiting goblet cell proliferation.^24^, ^25^ Xiang et al.^26^ demonstrated that IFN‐γ was dose‐dependent in the regulation of mucous cell metaplasia (MCM): when mice were exposed to allergens for a short period (5 days later), the presence of low concentrations of IFN‐γ seemed to enhance MCM through the STAT1 signaling pathway. However, IFN‐γ concentrations increase with long‐term exposure to allergenics (after 10 days), and high concentrations of IFN‐γ may inhibit IL‐9 ‐ and IL‐13‐induced MCM processes by affecting bax activation levels.^26^ In human bronchial epithelial cells, IFN‐γ negatively regulates MUC5AC production at the transcriptional level, that is, it blocks the binding of Sp1 to the MUC5AC promoter region via the JAK1/STAT1 pathway, and thus inhibits transcription of the *MUC5AC* gene.^27^ In CRS, IFN‐γ blocks the mucin‐production process in goblet cells in two ways. On the one hand, IFN‐γ blocks the release of calcium ions stimulated by cholinergic agonists via the JAK1/STAT1 signaling pathway, which directly reduces mucin secretion. On the other hand, IFN‐γ inhibits goblet cell proliferation and indirectly reduces mucin production by affecting the STAT1 signaling pathway.^28^, ^29^, ^30^ Jiao et al.^31^ observed that IFN‐γ could reduce the ciliate cell differentiation in patients with CRSwNP; however, the numbers of goblet cells did not significantly increase after IFN‐γ stimulation, and mucin secretion did not significantly change. These outcomes may have been related to individual and regional differences among the selected participants.^31^ In addition, in triple‐negative breast cancer, IFN‐γ can induce *MUC1* gene expression by activating the JAK1/STAT1/IRF1 pathway, but no similar study has been conducted in CRS.^32^ The above findings suggest that IFN‐γ can negatively regulate mucin secretion in a type 1 immune response of CRS using either its direct or indirect effects.

#### 2.1.2 TNF‐α and mucin

TNF‐α is also a typical Th1 cytokine that is closely related to a type 1 immune response, and exists in the form of a transmembrane protein on the surface of various inflammatory cells (i.e., T lymphocytes, natural killer cells, and macrophages). TNF‐α is involved in a variety of signal transduction pathways, exerts pleiotropic effects that regulate nuclear transcription factors, and can mediate and regulate different target genes in a variety of cell types.^33^ Polymorphism of the *TNFα‐1031* gene might be significantly correlated with the incidence of atopic CRS.^34^ Peter et al.^35^ classified patients with CRS treated with TNF‐α inhibitors into a separate subtype and also found that CRS on TNF‐α inhibitors (TNFαis) significantly reduced the number of eosinophils when compared to patients with CRSwNP.

Many studies have also shown that TNF‐α is involved in regulating mucin production and secretion in human airway epithelial cells (Figure 1). For example, Lee et al.^36^ found that TNF‐α could induce expression of the *MUC5AC* gene via nuclear translocation of the p65 subunit in NF‐κB induced by TNF‐α in human respiratory epithelial cells, and that p65 was a transcription factor that regulated *MUC5AC* gene expression.^37^ Song et al.^38^ proposed the following mechanism by which TNF‐α might induce *MUC5AC* gene expression: TNF‐α activates mitogen, mitogen‐ and stress‐activated protein kinase 1(MSK1), cAMP response element‐binding protein, and cAMP response element through ERK1/2 and p38MAP kinases, thereby causing a signaling cascade and inducing *Muc5ac* gene expression.^39^ In human lung mucoepidermoid cancer cell lines, TNF‐α mediates *MUC‐2* gene expression via the protein kinase C and tyrosine kinase signal transduction pathways in a concentration‐dependent manner.^40^, ^41^ However, with the increase in goblet cells, mucus gels are formed in the inflammatory region, and make it difficult for TNF‐α to bind with its receptors. This decrease in binding reduces the pro‐inflammatory effects of TNF‐α.^42^, ^43^ TNF‐α‐mediated mucin secretion can be accomplished through a synergistic action with other inflammatory factors. Yoon et al.^44^ found that TNF‐α could significantly increase the expression of MUC8 mRNA, whereas the levels of MUC5AC, MUC5B, and MUC2 mRNA expression did not increase in human nasal mucosal epithelial cells. However, when TNF‐α acts in conjunction with IL‐1β, MUC2 mRNA levels are increased.^45^ TNF‐α was shown to significantly increase the levels of MUC5AC and human chloride channel calcium‐activated 1 (hCLCA1) protein in primary epithelial cells derived from sinus mucosa, and MUC5AC expression was inhibited by chlorine channel blockers, indicating that TNF‐α can increase the expression of mucin in CRS by directly up‐regulating hCLCA1.^46^, ^47^, ^48^ At the level of gene regulation, Lee et al.^49^ suggested that TNF‐α regulates *MUC5AC* gene expression in human nasal epithelial cells via the NF‐κB mediated signaling pathway.^50^ The decreased expression of MUC1 seen in patients with CRSwNP may be related to impaired TNF‐α‐mediated GR‐α nuclear translocation and inhibition of p‐p65 expression.^51^ However, in human alveolar cells, TNF‐α can induce *MUC1* gene transcription and increase MUC1 expression by activating the MEK1/2‐related signaling pathway.^52,53^In guinea pig trachea epithelial cells, TNF‐α can promote the hypersecretion of mucin such as MUC5AC by activating PLC‐related signaling pathways.^54^, ^55^, ^56^

### 2.2 Regulation of mucin by Th2 cytokines associated with a type 2 immune response

#### 2.2.1 IL‐4/IL‐13 and mucin

**FIGURE 2:**
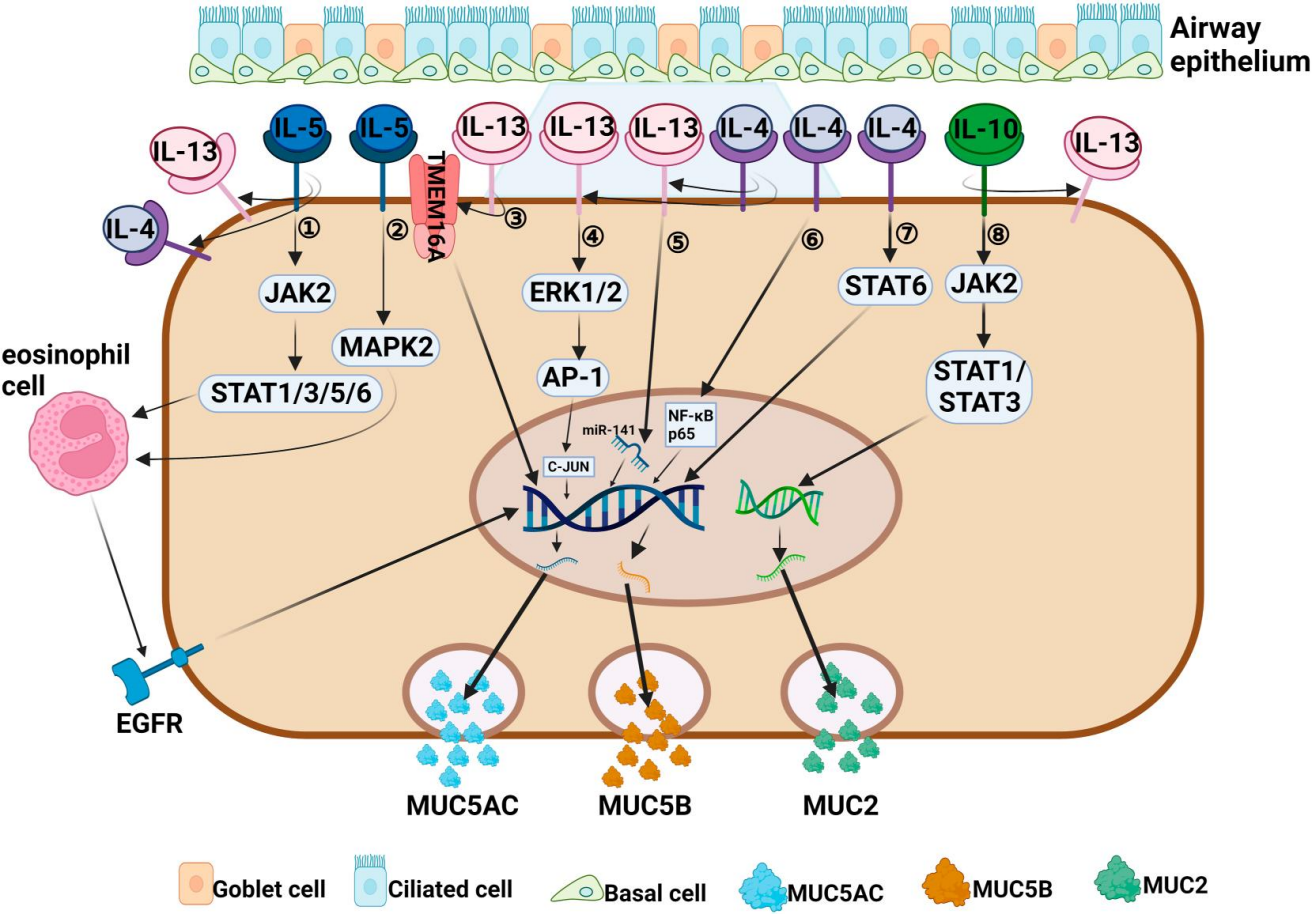
Regulation of mucin by Th2 cytokines associated with a type 2 immune response. (1, 2) IL‐5 can activate eosinophils through the activation of JAK2/STAT1/3/5/6 and MAPK2 signaling pathways,^92^, ^93^ which can cause the EGFR cascade reaction to induce *MUC5AC* gene transcription and protein synthesis.^95^, ^96^ IL‐5 can indirectly promote secretion of MUC5AC and MUC5B by enhancing the proinflammatory effect of IL‐4 and IL‐13.^62^ (3) IL‐13 regulates the production of MUC5AC by enhancing the action of membrane protein TMEM16 A.^64^, ^65^, ^66^ (4) IL‐13 enhances the action of C‐JUN to induce MUC5AC secretion by activating ERK1/2/AP‐1 signaling pathway.^67^ (5) IL‐13 promotes the production of MUC5AC by targeting miR‐141.^68^ (6, 7) IL‐4 can promote the production and secretion of MUC5AC and MUC5B by activating two signaling pathways of NF‐κB p65^72^ and STAT6.^70^ IL‐4 receptor can enhance the affinity of IL‐13 to IL‐13 receptor α1, indirectly leading to MUC5AC secretion.^62^. (8) IL‐10 induces *Muc2* gene expression through activation of JAK2/STAT1/STAT3 signaling pathway.^102^, ^103^ IL‐10 can indirectly induce MUC5AC secretion by enhancing the inflammatory process of IL‐13.^106^

CRSwNP involved in a type 2 inflammatory response is often accompanied by significant eosinophil infiltration.^57^, ^58^ IL‐4 and IL‐13 are typical Th2 cytokines involved in a type 2 immune response, which can lead to infiltration and recruitment of eosinophils, mast cells, and basophils, and also promote tissue remodeling, goblet cell proliferation, and mucus production (Figure 2).^59^, ^60^ IL‐13, IL‐4Rα, IL‐13Rα1, and MUC5AC were found to be highly expressed in nasal polyp tissue from patients with eosinophilic chronic rhinosinusitis (ECRS), and IL‐13 expression was positively correlated with MUC5AC expression, indicating that IL‐13 can directly regulate mucin hypersecretion.^61^ IL‐4 and IL‐13 share a common receptor signaling system, and MUC5AC and MUC5B secretion is significantly increased in CRSwNP characterized by tissue expression of IL‐4 and IL‐13.^62^, ^63^ IL‐4Rα is widely expressed in epithelial goblet cells and mucous cells of submucosal glands, and plays a role in airway inflammation by directly binding with IL‐4 or enhancing the affinity of IL‐13 for IL‐13Rα1, resulting in mucin secretion.^61^ IL‐13 can also promote MUC5AC secretion in human nasal polyp epithelial cells (HNPECs) via calcium‐activated chloride channels, such as transmembrane protein 16A (TMEM16 A) and CLCA.^64^, ^65^, ^66^ It was later found that in HNPECs, IL‐13 activates the extracellular signal‐regulated kinase (ERK) 1/2 signaling pathway and the downstream AP‐1 related gene *C‐JUN* by binding to IL‐13 receptor α2 (IL‐13Rα2), and inducing goblet cell proliferation and MUC5AC secretion.^67^ A recent study found that miR‐141 regulates the process of IL‐13‐induced production of MUC5AC in normal tracheobronchial epithelial cells of asthmatic patients, and may be involved in the transformation of basal cells into mucus‐secreting goblet cells.^68^ IL‐4 can maintain the production of constitutive IL‐4 and other key T2 cytokines in Th2 cells via IL‐4Rα signaling, and become rapidly upregulated in mucosal inflammatory states.^62^, ^69^ Significantly up‐regulated expression of MUC5AC was found in lung tissue of IL‐4 transgenic mice.^70^, ^71^ IL‐4 may regulate the expression of MUC5AC by inducing the phosphorylation of STAT6,^70^ and may also promote the synthesis of MUC5AC by inducing Ca^2+^ influx and NF‐κB p65 pathway.^72^ In addition, IL‐4 can also improve the production and transport speed of mucin.^73^, ^74^

In addition, the regulation of mucin by IL‐4 and IL‐13 can be mediated and amplified in response to certain other inflammatory factors (Figure 2). For example, IL‐31 is a four‐helix bundle cytokine derived from T‐helper lymphocytes, mainly produced by Th2 cells.^69^ In patients with CRSwNP, the expression of IL‐31 is significantly increased and positively correlated with the expression of Th2 cytokines (IL‐4, IL‐13), which can mediate and amplify Th2 inflammation.^75^ IL‐31 synergizes with IL‐4 or IL‐13 to induce *MUC5AC* gene expression in HM3‐MUC5AC cells and human airway A549 cells.^76^ IL‐33, an airway epithelium‐derived nuclear cytokine belonging to the IL‐1 family, is constitutionally expressed in the epithelial cells of nasal polyps.^77^ In the presence of IL‐33, Th2 cells can synthesize more IL‐5 and IL‐13, which is a key activator driving type 2 immune response.^78^ Hajime et al.^79^ found that IL‐33 can induce mucin gene expression and goblet cell proliferation in human nasal epithelial cells, and the specific mechanism remains to be further studied. IL‐6 is a multifunctional B‐cell differentiation factor that can promote Th2 cell differentiation and is produced by various types of cells, such as T cells, B cells, monocytes, etc.^80^ IL‐6 can induce the pathologic and physiological process of CRSwNP,^81^ regulate the expression of IL‐13 in human lung epithelial cells, thus inducing airway mucous cell metaplasia, or directly induce the expression of *Muc5ac* and *Muc5b* genes in vitro.^82^, ^83^ In addition, IL‐25 (also known as IL‐17E) is a cytokine belonging to the IL‐17 family that can be produced by Th2 cells and plays an important role in promoting Th2‐mediated inflammation.^84^ Studies have shown that IL‐25 can directly promote the increase in MUC5AC expression by activating hypoxia‐inducing factor 1α,^85^ and indirectly promote the secretion of MUC5AC and MUC5B by promoting the production of IL‐4, IL‐5, IL‐13.^86^

#### 2.2.2 IL‐5 and mucin

IL‐5 is mainly produced by Th2 cells and ILC2 cells, and partially by mast cells and natural killer cells. IL‐5 is crucial for the differentiation, proliferation, migration, and activation of eosinophils.^87^, ^88^ IL‐5 levels are elevated in patients with CRSwNP and are associated with disease severity, comorbidities, prognosis, and treatment response.^89^, ^90^, ^91^ Zhang et al.^62^ found that the significant increase in MUC5AC and MUC5B secretion is not caused by a direct effect of IL‐5, but is closely related to the high expression of IL‐4, IL‐13, and IL‐4 receptors in IL‐5‐positive nasal polyps. It has been suggested that IL‐5 can bind to a specific IL‐5 receptor (IL‐5R) to drive eosinophilic chemotaxis, and activation via the JAK2‐STAT1/3/5/6 and Ras/mitogen‐activated protein kinase 2 signaling pathways.^92^, ^93^, ^94^ IL‐5‐activated eosinophils induces *MUC5AC gene* transcription and protein synthesis in human airway epithelial cells via the epidermal growth factor receptor (EGFR) cascade.^95^, ^96^ Therefore, it is reasonable to believe that IL‐5 can establish a Th2 inflammatory environment that promotes excessive mucin secretion (Figure 2).

#### 2.2.3 IL‐10 and mucin

IL‐10 is a potent anti‐inflammatory Th2 cytokine produced by a variety of cell types (including T helper cells, B cells, cytotoxic T cells, and natural killer cells).^97^, ^98^ IL‐10 level was lower in patients with CRSwNP and CRSsNP than that in healthy controls,^99^ while another study showed no significant difference in IL‐10 level in nasal mucosal tissue between CRSwNP and control subjects.^100^ Since these studies involved the patients from different regions, Xuan et al.^101^ attributed the difference in these findings to immunologic heterogeneity of CRS patients in different regions.

IL‐10 prevents protein misfolding via STAT1 and STAT3 signal transduction pathways, and thereby maintains mucin production in goblet cells and integrity of the mucus barrier (Figure 2).^102^, ^103^ IL‐10‐sensitized mice display increased mucin production in airway epithelium after infection with RSV.^104^, ^105^ Lee et al.^106^ demonstrated that IL‐10 can induce goblet cell proliferation and enhance mucin gene expression to induce airway diseases in transgenic mice with overexpression of IL‐10 in the lungs. Specifically, the induction effect of IL‐10 on mucin is partially mediated by an IL‐13‐dependent pathway (IL‐13/IL‐4Rα/STAT‐6 pathway).^106^ The other mediation method is by enhancing *Gob‐5* gene expression, which induces mucogenesis in the airway.^106^, ^107^ Whether IL‐10 is involved in the regulation of mucin secretion in CRS through the above mechanism remains to be further studied.

### 2.3 Regulation of mucin by Th17 cytokines associated with a type 3 immune response

#### 2.3.1 IL‐17A and mucin

**FIGURE 3:**
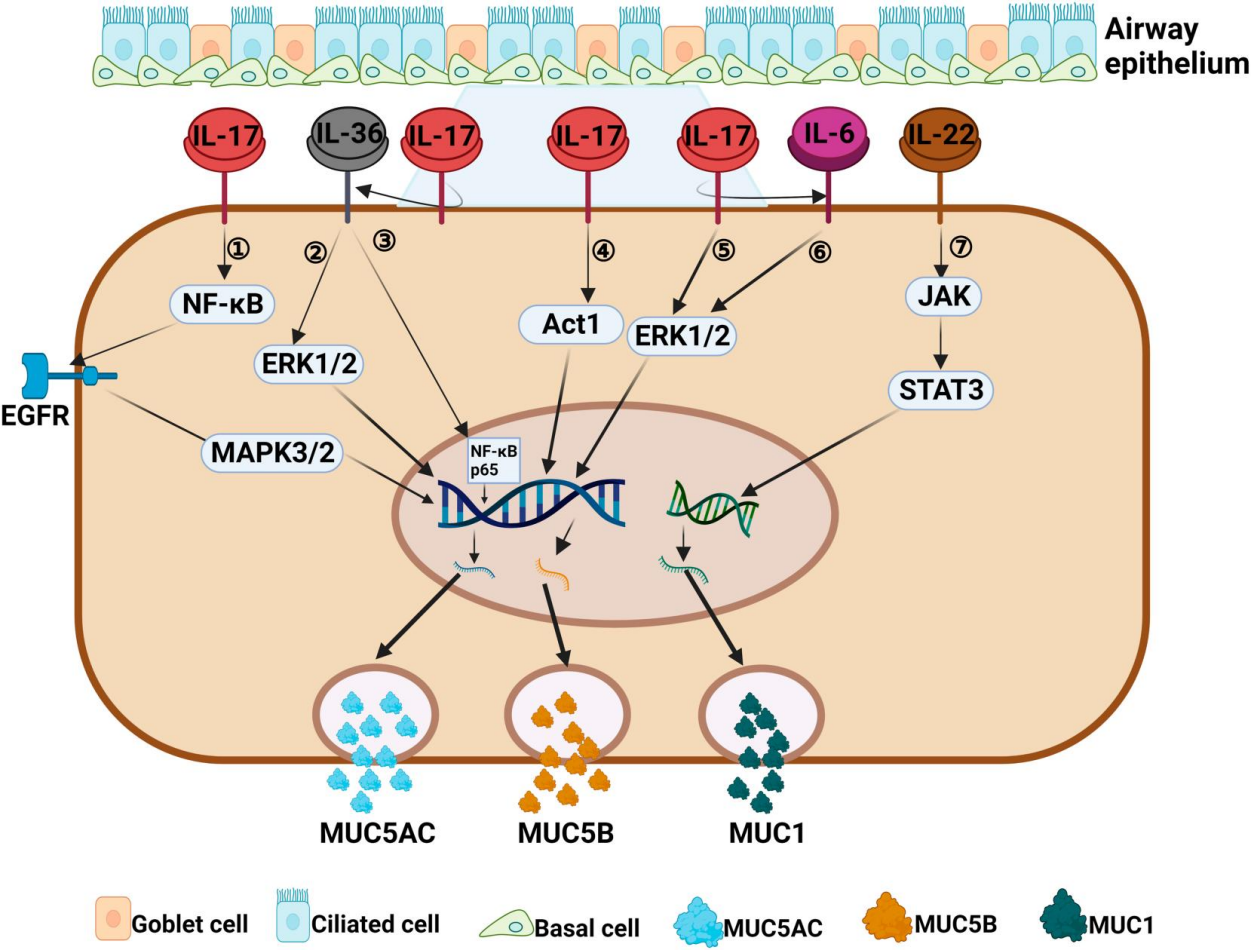
Regulation of mucin by Th17 cytokines associated with a type 3 immune response. (1) IL‐17 can induce MUC5AC secretion by activating the NF‐κB/EGFR/MAPK3/2 signaling pathway.^116^, ^117^, ^118^ (2, 3) IL‐17 can indirectly upregulate the expression of *MUC5AC* gene by IL‐36‐mediated ERK1/2 or NF‐κB p65 signaling pathway.^119^, ^120^ (4) IL‐17 can induce the expression of *MUC5AC* gene by activating Act1 signaling pathway.^114^ (5, 6) IL‐17 can directly or indirectly (via IL‐6) activate the ERK1/2 signaling pathway to induce MUC5B secretion.^111^, ^112^ (7) IL‐22 can induce MUCI secretion by activating JAK1/STAT3 signaling pathway.^126^, ^127^

While IL‐17A is mainly secreted by Th17 cells, it is also secreted by lymphoid tissue‐induced cells, congenital type 3 lymphocytes (ILC3), and natural killer cells.^108^, ^109^ IL‐17A can induce the expression of chemokines and recruit lymphocytes, neutrophils, and macrophages.^110^ IL‐17A is closely related to the function of CRS airway mucocilia and can regulate mucin expression (Figure 3).^31^ Our recent research showed that IL‐17A, IL‐17AR, and MUC5AC are highly expressed in nasal tissues from non‐ECRS patients when compared to ECRS patients, and IL‐17A is positively correlated with MUC5AC expression,^61^ indicating that IL‐17A plays a key role in mucin hypersecretion in a type 3 immune response. Chen et al.^111^ reported that IL‐17 directly regulates *MUC5B* gene expression at the promoter level via the ERK1/2 signaling pathway or via IL‐6 paracrine or autocrine loops in primary human tracheobronchial epithelial cells.^112^ Fujisawa et al.^113^ suggested that IL‐17A is able to promote MUC5AC secretion through its effects on NF‐κB‐based transcriptional mechanisms in airway epithelial cells. Later, Xia et al.^114^ demonstrated that IL‐17A can also induce MUC5AC expression via the Act1‐mediated signaling pathway by interfering with siRNA.^115^ Furthermore, IL‐17 can upregulate metalloproteinase‐9 (MMP‐9) expression via the NF‐κB pathway in nasal epithelial cells,^116^ and induce MUC5AC secretion via the epidermal growth factor receptor (EGFR)‐ mitogen‐activated protein kinase 3/2 (MAPK3/2) pathway.^117^, ^118^

In addition, IL‐17A has been shown to modulate IL‐36γ secretion in nasal epithelial cells, and evidence shows that the IL‐17A‐IL‐36γ axis mediates positive cross‐talk between epithelial cells and neutrophils in CRSwNP.^119^ IL‐36γ induces MUC5AC expression in human nasal epithelial cells via the ERK1/2 and NF‐κB p65 pathways upon binding to the IL‐36 receptor.^120^

#### 2.3.2 IL‐22 and mucin

IL‐22 is a cytokine in the IL‐10 family, and is mainly produced by Th17 cells and innate lymphocytes. IL‐22 can induce cell proliferation and inhibit cell apoptosis.^121^ Relevant studies have shown that the levels of IL‐22 and its receptor (IL‐22R1) in invasive inflammatory cells and epithelial cells of the nasal passages of patients with CRSwNP are significantly increased. IL‐22 levels are also closely related to the severity of clinical symptoms and difficulty of treating CRSwNP.^19^, ^122^, ^123^, ^124^ IL‐22 can enhance the expression of MUC1 (a membrane‐bound mucin located on the nasal mucosa) in nasal polyp tissues via an IL‐22R1 dependent pathway (Figure 3).^125^ In addition, IL‐22 can promote the restitution of mucin‐producing goblet cells by activating STAT3. The most common way in which IL‐22 acts is to induce activation of the JAK1/STAT3 pathway and phosphorylation of Jak1 and Tyk2.^128^, ^129^, ^130^, ^131^ Whether these IL‐22‐mediated pathways can promote mucin secretion requires further investigation.

### 2.4 The role of Th cytokines‐related biologics in the treatment of mucous hypersecretion in CRS

So far, some monoclonal antibodies in CRSwNP have entered clinical application or clinical trial stage to treat nasal polyps, including omalizumab (anti‐IgE), reslizumab (anti‐IL‐5), mepolizumab (anti‐IL‐5) and dupilumab (anti‐IL‐4Rα).^132^ These biologics can effectively improve patients' symptoms such as runny nose, nasal obstruction, dysmorphia, and endoscopic polyp size, and inhibit the expression of IgE, IL‐5, IL‐4, and IL‐13.^133^, ^134^ Mucin is the most important solid component of mucus, and excessive secretion of mucus is an important cause of abnormal nasal discharge and nasal congestion. We have reason to believe that these biologics might reduce the abnormal secretion of mucin by inhibiting type 2 inflammation. Currently, there are no monoclonal antibodies targeting inflammatory cytokines of type 1 and type 3 to treat patients with CRS. Some studies have shown that IL‐17A can enhance the expression of type 2 cytokines and eosinophil infiltration in ECRS or NP mouse model, which might provide a new option for the treatment of CRS with biologics.^135^, ^136^

## 3 SUMMARY

The mucociliary clearance system is an important barrier that protects the upper respiratory tract in normal nasal mucosal epithelium. The normal secretion of mucin is an important factor for the maintenance of normal airway function. An abnormally high level of mucin secretion and insufficient mucin clearance in CRS leads to mucus accumulation, which in turn leads to upper airway obstruction and infection and aggravates airway inflammation. We believe that the main Th cytokines associated with specific immune responses play a key role in regulating abnormal mucin secretion in different endotypes of CRS. Specifically, IFN‐γ exerts negative regulatory effects on mucin secretion by reducing goblet cell metaplasia or directly inhibiting mucin expression. In contrast, TNF‐α, IL‐4, IL‐5, IL‐13, IL‐10, IL‐17, and IL‐22 can exert positive regulatory effects on mucin by affecting various signaling pathways. The identification of the mechanisms by which Th cytokines affect mucin secretion in different endotypes of CRS might provide precise intervention targets for the treatment of airway mucus overproduction, and thereby improve the quality of life of CRS patients.

## 4 OUTLOOK

Above, we summarized the mechanisms by which Th cytokines secreted by typical Th1,Th2 and Th17 cells regulate mucin secretion in CRS. At the same time, some other Th cell subsets (e.g., Th9, Th22, Treg cells, and Tfh cells) secrete different cytokines (IL‐9, IL‐21, IL‐2, etc.), which are also involved in the pathogenesis of CRS^131^, ^137^, ^138^ and have certain regulatory effects on mucin secretion. For example, IL‐9, as a relevant cytokine in type 2 CRSwNP, stimulates MUC5AC expression through hCLCA1, and regulates *MUC2* and *MUC5AC* gene transcription by activating the IL‐9 receptor on the airway epithelium.^47^, ^139^ Additional studies are needed to explore the specific pathophysiological mechanisms of mucin regulation in multiple Th inflammatory patterns. The results of those studies can be used to guide the further treatment of airway diseases with mucin hypersecretion by precisely blocking certain cytokine receptors or their activated signaling pathways.

## AUTHOR CONTRIBUTIONS

**Zhaoxue Zhai**: Conceptualization (equal); Investigation (equal); Methodology (lead); Resources (equal); Writing – original draft (lead). **Liting Shao**: Data curation (equal); Investigation (equal); Methodology (equal); Resources (equal); Writing – original draft (equal). **Zhaoyang Lu**: Data curation (equal); Investigation (equal); Methodology (equal); Project administration (equal); Resources (equal). **Yujuan Yang**: Data curation (supporting); Methodology (supporting); Resources (supporting); Visualization (supporting); Writing – original draft (supporting). **Jianwei Wang**: Methodology (supporting); Resources (supporting); Visualization (supporting). **Zhen Liu**: Data curation (supporting); Investigation (supporting); Methodology (supporting); Resources (supporting). **Huikang Wang**: Data curation (supporting); Investigation (supporting); Project administration (supporting). **Yang Zheng**: Project administration (supporting); Resources (supporting); Visualization (supporting). **Haoran Lu**: Data curation (supporting); Investigation (supporting); Resources (supporting). **Xicheng Song**: Conceptualization (lead); Funding acquisition (lead); Project administration (lead); Writing – review & editing (lead). **Yu Zhang**: Conceptualization (lead); Funding acquisition (lead); Investigation (equal); Project administration (lead); Supervision (lead); Writing – review & editing (lead).

## CONFLICT OF INTEREST STATEMENT

The authors declare no conflicts of interest.

## Data Availability

The data that support the findings of this study are available from the corresponding author upon reasonable request.
